# Metadynamics-Based Approaches for Modeling the Hypoxia-Inducible Factor 2α Ligand Binding Process

**DOI:** 10.1021/acs.jctc.1c00114

**Published:** 2021-06-04

**Authors:** Lara Callea, Laura Bonati, Stefano Motta

**Affiliations:** Department of Earth and Environmental Sciences, University of Milano-Bicocca, Piazza della Scienza 1, 20126 Milan, Italy

## Abstract

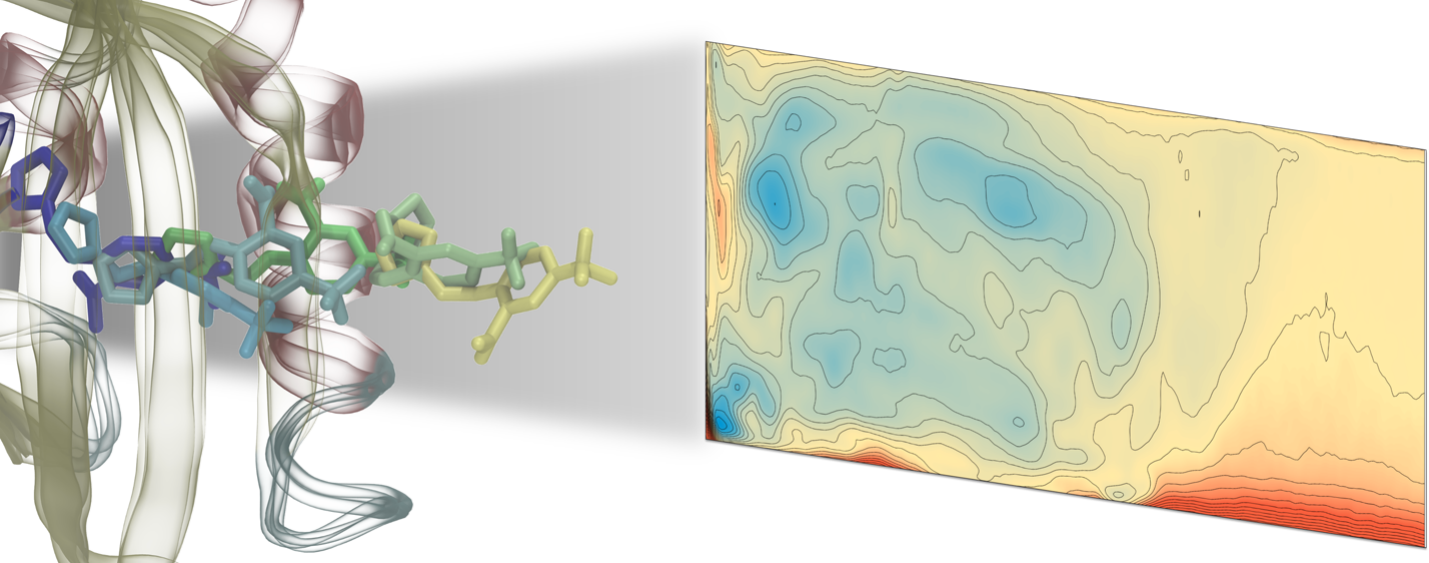

Several methods based on enhanced-sampling molecular dynamics have been proposed for studying ligand binding processes. Here, we developed a protocol that combines the advantages of steered molecular dynamics (SMD) and metadynamics. While SMD is proposed for investigating possible unbinding pathways of the ligand and identifying the preferred one, metadynamics, with the path collective variable (PCV) formalism, is suggested to explore the binding processes along the pathway defined on the basis of SMD, by using only two CVs. We applied our approach to the study of binding of two known ligands to the hypoxia-inducible factor 2α, where the buried binding cavity makes simulation of the process a challenging task. Our approach allowed identification of the preferred entrance pathway for each ligand, highlighted the features of the bound and intermediate states in the free-energy surface, and provided a binding affinity scale in agreement with experimental data. Therefore, it seems to be a suitable tool for elucidating ligand binding processes of similar complex systems.

## Introduction

Understanding the thermodynamic principles behind the mechanism of ligand-protein binding is very important for the development of a successful drug design campaign. Experimental techniques are able to estimate binding thermodynamic and kinetic properties but cannot provide the atomistic insight that forms the basis of rational approaches to drug design. In this context, *in silico* methods are becoming increasingly effective in complementing experiments and providing atomic-level descriptions of ligand binding. Docking methods are widely used to rapidly screen libraries containing thousands or even millions of compounds^1^ but suffer from several limitations, one above all the lack of protein flexibility. In cases where the induced-fit effects of the ligand are important, a number of alternative methods have been proposed to account for protein flexibility in ligand binding.^2,3^ For example, flexibility can be introduced by docking ligands to an ensemble of different protein conformations (ensemble docking).^4−7^ This method only partially introduces protein flexibility, and results strongly depend on the type of ensemble used. Recently, molecular dynamics (MD) simulations have been used to study processes happening on timescales that range from nanoseconds to milliseconds and beyond,^8^ making them attractive for the study of ligand binding. However, computation of key thermodynamic quantities requires the observation of multiple binding events to obtain reliable statistics on the process, thus increasing the computation time. Typically, enhanced-sampling techniques are used to speed up the simulation of the binding/unbinding events.^9,10^ Most of these techniques make use of a bias potential that forces the system to sample higher-energy regions, speeding up the crossing of energy barriers.

Among the methods for studying ligand binding based on enhanced-sampling MD,^11−17^ in this work, we focused on steered MD^18^ (SMD) and metadynamics^19^ (MetaD).

SMD was inspired by single-molecule pulling experiments and applies a moving restraint bias that pulls the system along a selected variable. Despite its wide applications to the study of (un)folding mechanisms of proteins^20,21^ and transportation of ions and other molecules across membrane channels,^22,23^ SMD has also emerged as a method for studying ligand (un)binding,^24−27^ given that it is particularly well-designed for the investigation of entry and exit pathways. Its points of strength are the easy setup and the shortness of simulations.^28^ On the other hand, SMD still suffers from several limitations, in particular regarding the calculation of the potential of the mean force (PMF).^28^ During the pulling, indeed, a part of the work is spent as dissipative work, and convergence can be difficult to reach. In theory, the Jarzynski equality may account for the dissipative part of the work; however, when the range of work obtained in multiple replicas is broad, simulations with the lowest work contribute the most to the calculation of the average work.^29^ These limitations may be overcome by performing a large number of replicas and reducing the pulling speed, but for some complex systems, this is often not enough.

MetaD is a method based on the introduction of a history-dependent bias potential applied to a small number of suitably chosen collective variables (CVs).^19,30,31^ Within the CV subspace, the potential is built up by adding Gaussians along the sampled trajectory to discourage the system from revisiting already sampled configurations. The Gaussian height, the Gaussian width, and the deposition time are crucial parameters to obtain a converged free-energy surface (FES).^30^ However, the choice of the CVs is the most critical aspect in MetaD, and results can be seriously affected by the omission of important degrees of freedom (hysteresis).^30^ Given that the computational cost to reconstruct the free-energy surface exponentially grows with the number of CVs, Branduardi et al.^32^ developed the path collective variable (PCV) method, which allows exploration of complex multidimensional processes along a predefined pathway described by a single CV. An additional CV, which describes the distance from the reference path, usually completes the set of CVs necessary to efficiently sample the process of interest. In the past few years, MetaD in its various forms has been successfully applied to several ligand-protein binding studies.^33−41^ One of the main advancements in this field was the development of the so-called “funnel metadynamics” where a funnel-shaped potential limits the space available to the ligand once it has undocked.^42,43^

Here, we investigate the ligand binding process to the hypoxia-inducible factor 2α (HIF-2α), a pharmaceutically relevant system widely recognized as a target for cancer therapy.^44^ HIF-2α mediates the physiological responses to hypoxia through heterodimerization with the aryl hydrocarbon receptor nuclear translocator (ARNT).^45,46^ Both the HIF-2α and ARNT belong to the mammalian basic helix–loop–helix-PER-ARNT-SIM (bHLH-PAS) family of proteins, where members modulate transcriptional responses to environmental and cellular signals and are involved in a variety of physiological processes and diseases in humans.^44,47^ Members of the bHLH-PAS family present an N-terminal bHLH region for DNA binding, two PAS domains (PAS-A and PAS-B) with the role of both sensing external signals and recognizing the dimerization partner and a transactivation domain. For a long time, only another bHLH-PAS protein, the aryl hydrocarbon receptor (AhR), was known to be activated by binding to a wide range of ligands within its PAS-B cavity.^48,49^ More recently, following the discovery of a buried cavity within the HIF-2α PAS-B domain,^50^ several artificial small molecules were identified as HIF-2α ligands and potential inhibitors of the HIF-2α:ARNT dimerization.^51−56^ The structural determination of the HIF-2α:ARNT dimer encompassing the whole bHLH-PAS region, in the unbound, DNA-bound, and inhibitor-bound forms,^45^ recently allowed us to investigate the inhibition mechanism of the 0X3 antagonist and to shed light on pharmacophoric features required for the development of new inhibitors.^57^

In this work, we combined SMD and PCV MetaD simulations to investigate the binding process of two known ligands to the HIF-2α PAS-B domain. We were aimed at both investigating the ligand entrance pathway into the binding cavity and assessing the validity of the selected methods for such a complex system. In fact, the buried nature of the cavity makes it difficult to imagine the entry or exit route of the ligand, and although a previous MD investigation identified probable pathways for water exchange with the bulk solvent,^53^ access of larger organic molecules to the cavity has never been studied. Moreover, it is conceivable that ligand entrance into this cavity may involve significant protein conformational rearrangements. The above features of the system make simulation of the ligand binding process a nontrivial task and required the development of specific methodological approaches. In light of the obtained results, these methods appear to be suitable also for the elucidation of other ligand binding processes with similar characteristics.

## Methods

### System Preparation and Molecular Dynamics Simulation

Crystal structures of HIF-2α in its bound state with the THS-020 ligand (PDB ID: 3H82(53)) were obtained from the Protein Data Bank (PDB).^58^ The PAS-B of the ARNT protein partner, included in the X-ray deposition, was removed. This does not induce perturbations in the structure of the HIF-2α PAS-B, as shown by the RMSD plot (Figure S1) that highlights the stability of the HIF-2α domain during the MD simulation. The KG-721 bound form was obtained with molecular docking calculations (see the next subsection). The protein was prepared with the Protein Preparation Wizard^59^ included in Maestro: hydrogen atoms were added, all water molecules were removed, C- and N-terminal cappings were added, disulfide bonds were assigned, and residue protonation states were determined by PROPKA^60^ at pH = 7.0. The ligands were prepared using the LigPrep^61^ tool included in Maestro in order to optimize the structures. The partial charges of ligands were calculated using the RESP^62^ method at the AM1-BCC^63^ level of theory in Antechamber,^64^ while a GAFF^65^ parametrization was used to achieve the complete topological description of each ligand. The unbiased MD simulations were performed using GROMACS 2018.6.^66^ The protein was solvated in an orthorhombic box with TIP3P^67^ water molecules and neutralized with Na^+^/Cl^–^ ions. The minimal distance between the protein and the box boundaries was set to 20 Å. The Amber ff14SB force field^68^ was used for the protein, and a multistage equilibration protocol was applied: the system was first subjected to 2000 steps of the steepest descent energy minimization, with positional restraints (239 kcal mol^–1^ nm^–2^) for the backbone and the ligand. Subsequently, a 200 ps NVT MD simulation was used to heat the system from 0 to 100 K with restraints lowered to 96 kcal mol^–1^ nm^–2^; then, the system was heated up to 300 K in 400 ps during an NPT simulation with further lowered restraints (48 kcal mol^–1^ nm^–2^). Finally, the system was equilibrated during an NPT simulation for 2 ns with backbone restraints lowered to 12 kcal mol^–1^ nm^–2^. In the NVT simulations, temperature was controlled using the Berendsen thermostat^69^ with a coupling constant of 0.2 ps, while in the NPT simulations, the V-rescale thermostat^70^ (coupling constant of 0.1 ps) was used and the pressure was set to 1 bar with the Parrinello–Rahman barostat^71^ (coupling constant of 2 ps). A time step of 2.0 fs was used, together with the LINCS^72^ algorithm to constrain all the bonds. The particle mesh Ewald method^73^ was used to treat the long-range electrostatic interactions with the cutoff distance set at 11 Å. Short-range repulsive and attractive dispersion interactions were simultaneously described by a Lennard-Jones potential, with a cutoff at 11 Å. Finally, a 20 ns production run was performed without the constraints.

### Molecular Docking of the KG-721 Ligand

Conformational analysis of the ligand structure was performed using Macromodel^74^ with the OPLS_2005^75^ force field. The obtained global minimum was used as the starting point for molecular docking calculations using Glide^76^ XP^77^ (Extra Precision). In particular, Glide uses a flexible ligand-rigid protein approach, in which a series of hierarchical filters are applied to find the possible positions and conformations of the ligand in the binding cavity (poses). The properties of the protein are represented on a grid that provides gradually more accurate scores. The initial screenings are deterministically performed over the complete phase space of the ligand to identify the most promising poses. From the selected poses, the ligand is then refined in the torsional space in the receptor field. To take into account the flexibility of the protein, the ensemble-docking approach was used, which involves ligand docking to multiple receptor conformations. These can be derived either experimentally or computationally (e.g., by MD simulations).^5^ The conformational ensemble here selected consisted of the crystallographic structures of the HIF-2α PAS-B in complex with artificial ligands available in PDB (3F1O,^50^3H82,^53^3H7W,^53^4GS9,^51^ and 4GHI(52)). The results showed that the best XP score is the one related to the KG-721 ligand in the 4GHI structure.

### Steered MD Simulations (SMD)

In SMD simulations,^26,27,78^ a time-dependent external force is applied to the ligand to aid its unbinding from the protein. In particular, the transition between the bound and the unbound states is achieved by adding a harmonic time-dependent potential, acting on a descriptor (or collective variable), to the standard Hamiltonian. During the transition, the external work performed on the system can be calculated using the Jarzynski equation.^79^ All the SMD simulations were performed using the PLUMED 2.4.6^80,81^ plugin integrated in GROMACS 2018.6.^66^ We chose the ligand-protein distance as the pulling variable. This was defined as the distance between the center of mass of selected atoms at the bottom of the binding cavity (different for the two pathways, see Figure S2) and the center of mass of the ligand heavy atoms. The spring constant was set to the value of 10.0 kcal/mol·Å^2^, and the ligand was pulled from the initial value of CV to 35 Å in 25 ns with a resulting pulling velocity of 0.984 Å/ns. We ran 50 independent replicas, and the time length for each simulation was 25 ns, which ensured the achievement of a complete solvation of the ligand in the unbound state. The starting point of each replica was derived from an ensemble of states extrapolated at regular time intervals of 0.2 ns from the last 10 ns of the unbiased simulation.

### Metadynamics (MetaD) and Path Collective Variables (PCVs)

The central idea of the metadynamics method^19,30^ is to bias the system along a set of CVs using a history-dependent potential. To achieve this, a Gaussian-shaped potential is added to bias the system at the current position of the CVs, at regular time intervals. This allows the system to escape from any local minimum and to visit new regions in the CVs space. In metadynamics, to push the system to visit even high free-energy regions, the Gaussian-shaped potential has constant height. On the contrary, in the well-tempered metadynamics^82^ approach, used in this work, the height of the Gaussian is decreased with the amount of bias already deposited according to
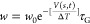
where *w*_0_ is an initial Gaussian height, Δ*T* an input parameter with the dimension of a temperature, *k*_B_ is the Boltzmann constant, and τ_G_ is the time interval at which Gaussians are deposited.^82^

The path CV formalism^32,83^ has been widely used to investigate biological processes, to compute their free-energy surfaces, and to characterize their kinetic behavior.^39,34^ In this work, PCVs were used to study the transition between the bound and the unbound states in the unbinding process of some HIF2-α ligands. We described the transition pathway with a set of frames derived from the SMD simulations: 12 frames were used for the THS-020 ligand and 11 frames for the KG-721 ligand. For the first part of the path, the frames (from 1 to 7 for THS-020 and from 1 to 6 for KG-721) were obtained from the SMD simulations with the lowest value of external work performed on the system. Frames were selected to be equally spaced (2 Å). For the second part of the path, the frames were obtained with linear interpolation (see the Supporting Information, Supplementary Text for the details). Following the procedure proposed by Branduardi et al.,^32^ we introduced two collective variables: *s*(*R*), the progress along the reference path, and *z*(*R*), the distance orthogonal to the reference path. The λ value was set to 33.0 nm^2^. The distance between the instantaneous conformational state during the simulation and the reference coordinates in the path was evaluated by the RMSD metric.^84^ In particular, the RMSD along the entry/exit pathway was calculated between a selection of protein atoms and all the ligand heavy atoms (see Figure S3). In all simulations, the Gaussian-shaped potentials were deposited every 500 simulation steps, the initial height was set to 1 kJ/mol, and the decay corresponding to a bias factor of 10 was chosen. The Gaussian widths (σ) for the *s*(*R*) and *z*(*R*) variables were set to 0.05 and 0.007, respectively. Widths were set so that they are about 1/3 of the CV standard deviations observed in the unbiased MD simulation. The two variables, *s*(*R*) and *z*(*R*), were constrained to be less than 12 and 0.2 nm^2^, respectively.

### Extraction of Minima and Cluster Analysis

To characterize the different minima identified on the final free-energy surface (FES), we extracted a group of frames belonging to each minimum hole. To obtain a representative structure of the complex in each minimum, a cluster analysis on the metadynamics trajectory frames with a stride of 10 ps was performed. The GROMOS^85^ clustering algorithm was applied, with a 2 Å RMSD cutoff on the heavy atoms of the ligands. The centroid of the most populated cluster was then defined as the representative structure in that minimum.

## Results and Discussion

**Figure 1:**
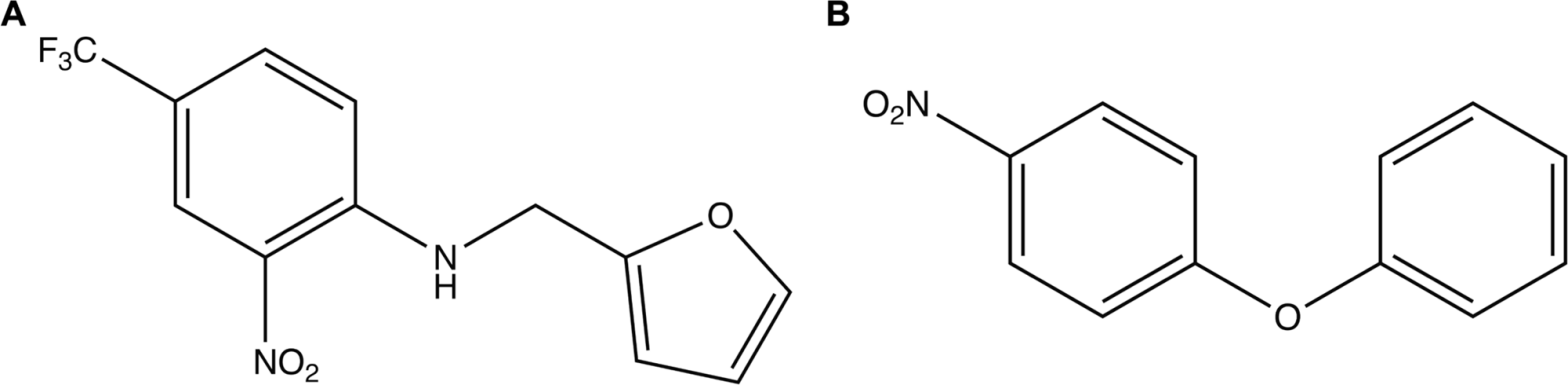
2D structure of THS-020 (A) and KG-721 (B).

The analysis of the unbinding pathways was performed for two of the HIF-2α ligands identified in the study of Key et al.^53^ The THS-020 ligand (Figure 1A) has a good binding affinity for the protein (Δ*G*_exp_ = −7.9 ± 0.5 kcal/mol), and the ligand-protein bound structure, determined by X-ray crystallography,^53^ is available. The KG-721 ligand (Figure 1B) is a lower-affinity ligand (Δ*G*_exp_ = −6.9 ± 0.1 kcal/mol) identified in the same work.^53^ We choose it among the other HIF-2α ligands,^50−56,86−88^ not only for the different binding affinity but also to deal with a molecule not congeneric to THS-020,^53,51^ with different physicochemical properties and with lower size. Moreover, for this ligand, no experimental structures of the ligand-protein complex are available, thus offering us the opportunity to study a system where the starting conformation, obtained by docking, could not take into account the induced-fit effects on the protein.

In the following two subsections, we present the application of a specific SMD MetaD protocol on the THS-020. We first used SMD to identify the unbinding pathway, and then, we applied PCV MetaD simulations to characterize the relevant states in the binding/unbinding process and to obtain a reliable estimate of the binding affinity. In the third subsection, we present the results obtained with the same protocol for KG-721.

### Identification of Unbinding Pathways for the THS-020 Ligand by the SMD Method

Starting from the X-ray structure for the HIF-2α PAS-B domain in complex with THS-020, the possible ligand unbinding pathways were investigated using the SMD approach. Steered molecular dynamics is a popular method for studying ligand-protein unbinding events^89−91^ and can provide both a qualitative description of the pathways and a quantitative estimate of the free-energy difference between the bound and unbound states.

To calculate the free-energy difference using the Jarzynski equality, it is necessary to have a large number of SMD replicas. To this aim, a 20 ns unbiased MD simulation was performed starting from the X-ray structure, generating an ensemble of 50 slightly different states of the complex (Figure S4a), extracted from the last 10 ns. These were then used as starting points for the 50 SMD simulations. The structural convergence of the unbiased simulation was assessed by calculating the RMSD matrix on the protein Cα atoms and on the ligand heavy atoms (Figure S4b). Each SMD replica was 25 ns long. To verify if the values chosen for the SMD simulation parameters (see the Methods section) are appropriate, the RMSD plot on Cα atoms (Figure S5) and the secondary structure conservation graphs (Figure S6) were calculated for the 50 replicas along path 1. As shown in Figure S6, no significant distortions of the protein structure (except for a slight deformation of the Fα helix upon ligand unbinding) were observed during simulations, thus confirming the validity of the proposed protocol.

**Figure 2:**
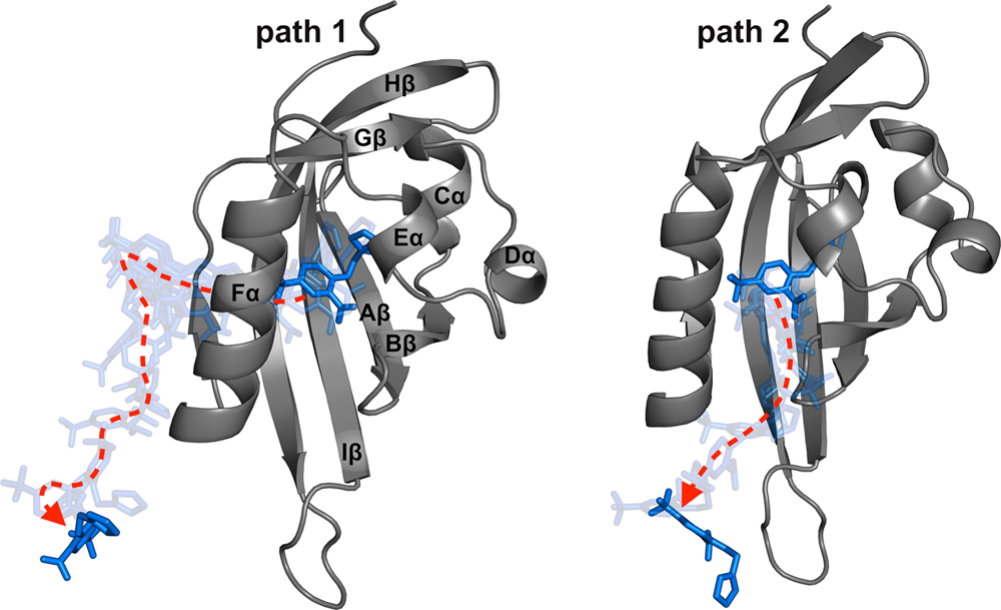
THS-020 unbinding pathways. In pathway 1 (left), the ligand passes through Fα and Gβ while in pathway 2 (right) through Fα, Eα, and the AB loop. The starting protein structure is represented as gray cartoons, the ligand conformations in the first and last frames of the trajectory as blue sticks, and the conformations of the ligand in the intermediate frames as transparent sticks.

Other authors identified two entry/exit pathways for solvent water by MD simulations of the apo HIF-2α PAS-B^53^: path 1 gets through the Fα helix and the Gβ strand, while path 2 gets through the Fα helix, the short Eα helix, and the AB loop (Figure 2). On this basis, for each of these two pathways, we calculated a CV allowing us to pull the ligand out of the binding cavity, by selecting an appropriate set of residues at the bottom of the binding cavity (see the Methods section).

The work profiles resulting from the 50 replicas, in Figure S7, show an increase in the work value during the initial part of the unbinding process followed by relatively settled work values, indicating the absence of interaction between the ligand and the protein.

All the curves show a similar profile, but a qualitative comparison of the total work reveals that less work is required for unbinding following pathway 1. Moreover, a broader range of values is observed for the replicas following pathway 2, indicating that higher barriers can occur in some of the replicas associated to this pathway. For a quantitative analysis, the minimum and maximum work values (*W*_min_ and *W*_max_), the minimum value of the maximal force (*F*_max_) among the replicas, and the free-energy difference between the unbound and the bound states (Δ*F*_unbind_) were extracted for each pathway (Table 1). The results show that the *W*_min_ necessary to pull the ligand outside the cavity along path 1 is about 10 kcal/mol less than that required for path 2; a similar trend is observed for the values of *W*_max_ and Δ*F*_unbind_. A difference of 75.59 pN between the *F*_max_ values in the two paths is observed, which confirms a clear preference of path 1 over path 2.

**Table 1:** Results of SMD Simulations for the THS-020 Ligand

pathway	*W*_min_ (kcal/mol)	*W*_max_ (kcal/mol)	*F*_max_ (pN)	Δ*F*_unbind_ (kcal/mol)	st. dev.
1	28.19	55.17	1013.77	30.55	16.80
2	38.16	66.42	1089.36	40.25	13.10

Therefore, steered MD allowed us to compare the two unbinding pathways of THS-020 and to select the preferred one by identifying a higher-energy barrier along pathway 2. However, SMD provided a value for the Δ*F*_unbind_, estimated by means of the Jarzynski equality, that was about 4 times higher than the experimental value. It is indeed known that these nonequilibrium simulations generally undersample the relevant protein-ligand states across the unbinding pathway, leading to errors in the computed binding free energy.^92^ Moreover, replicas with less work done on the system have an enormous weight compared to all the other trajectories, which makes the method extremely sensitive to insufficient sampling.^93^ For this reason, we then applied the PCV MetaD approach that was recently proposed as a valuable method for computing absolute binding free energies in ligand binding.^34,39,35^

### Metadynamics Simulations and Free-Energy Profiles with PCVs for THS-020

For a detailed mechanistic interpretation of the ligand binding/unbinding process, we used well-tempered metadynamics simulations with the PCV approach^83,35,94^ (see the Methods section). This allowed us to characterize the relevant states along the preferred path obtained with SMD simulations (the one with lower values of total work obtained from the SMD simulation, path 1), as well as to estimate the binding free-energy value. The key points of this method are the choice of appropriate CVs and the construction of a set of equally spaced frames along the CVs in terms of RMSD between adjacent snapshots. This frameset represents a reference path for investigating the process. As CVs, we used the progress along the path, *s*(*R*), and the distance orthogonal to the reference path, *z*(*R*). We want to underline the importance of the path construction phase, especially in a case with a buried binding site like the one presented in this work. Here, we decided to include both ligand and protein atoms in the frameset that represents the path to better describe the protein conformational changes during the process (mouth opening through side-chain conformational changes and small backbone adjustment). Only protein atoms involved in the conformational changes, highlighted by SMD simulations, were included. Moreover, a hybrid approach that combines frames from SMD simulations and linear interpolation was used for the inner and outer parts of the path, respectively. Details about the construction of the reference path can be found in the Supporting Information, Supplementary Text. The reference path obtained with this approach is represented in Figure S8. The RMSD matrix of the frameset (Figure S8) is a symmetric matrix with a typical gull wing shape, indicating that the frames are correctly equally spaced.

**Figure 3:**
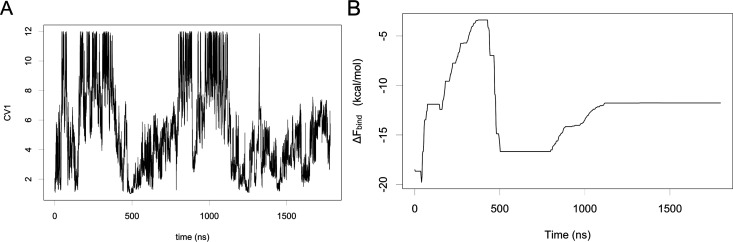
(A) Plot of the CV1 (*s*(*R*)) against simulation time during THS-020 MetaD simulation: the lowest values of *s*(*R*) correspond to the bound state while the highest to the unbound ones; (B) one-dimensional projection of the binding free-energy values associated to path 1 during the metadynamics simulation.

We collected a total of 1.8 μs of metadynamics simulation in which we observed several binding/unbinding events, as shown in Figure 3A. The binding free energy (Δ*F*_bind_), calculated as the free-energy difference between the deepest minima in the bound state and the flat plateau in the unbound state, turns out to be equal to −11.8 kcal/mol. The free-energy profile during the simulation, shown in Figure 3B, indicates that the simulation reaches a constant value of Δ*F*_bind_ after about 1200 ns. The convergence was also monitored by plotting the hill heights as a function of the simulation time (Figure S9).

**Figure 4:**
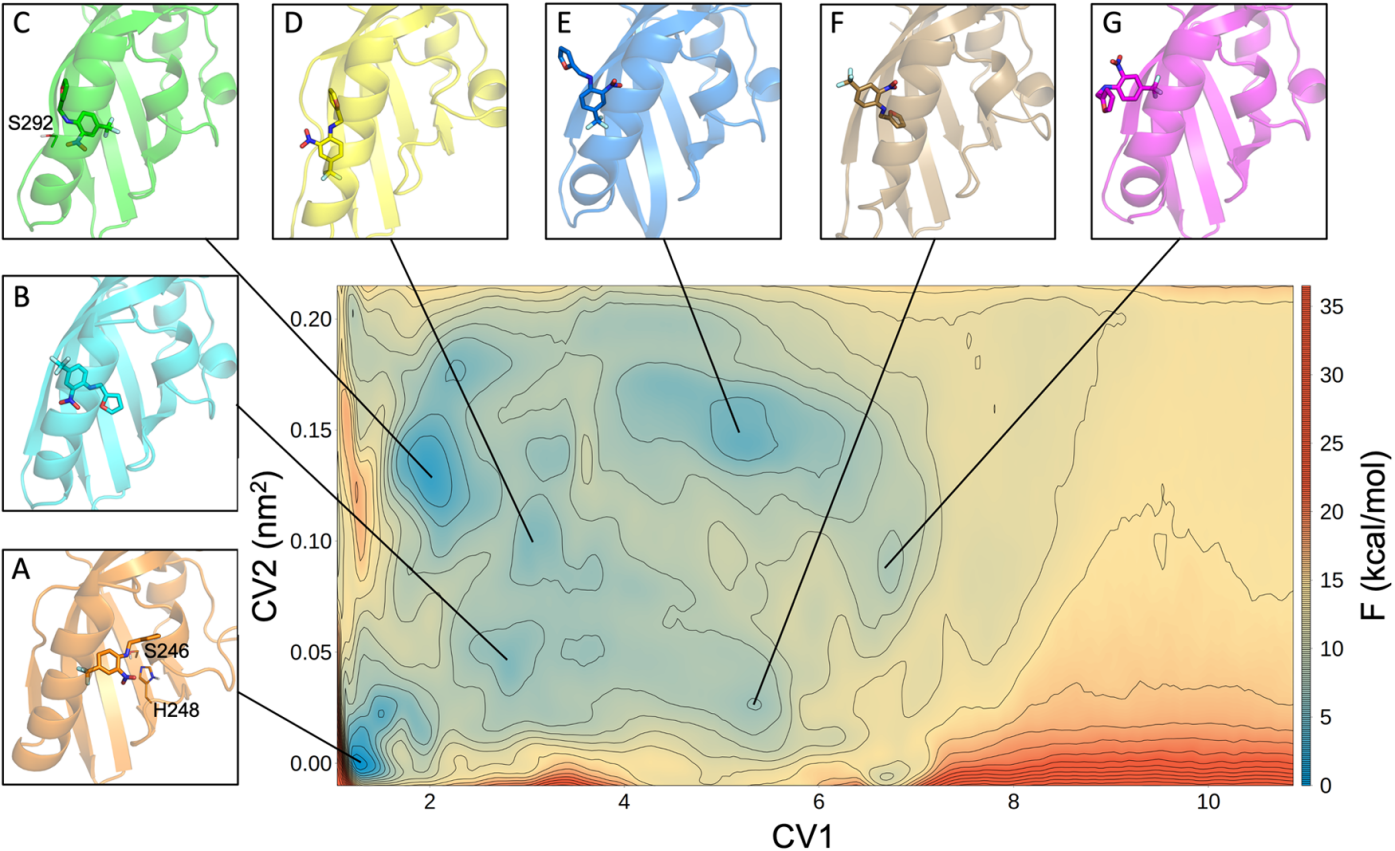
Free-energy surface obtained from the PCV approach for the binding/unbinding of the THS-020 ligand. The isolines are drawn using a 1.5 kcal/mol spacing. The 3D structures of the centroids of the main minima are reported with different colors: the protein is represented as cartoons and the ligand as sticks. The black lines indicate the corresponding minima in the FES.

The free-energy surface (FES) for the binding/unbinding process, as a function of CV1 (*s*(*R*)) and CV2 (*z*(*R*)) in 1.8 μs of metadynamics simulation, is shown in Figure 4 together with the relevant minima found along the pathway (labeled as A to G). The coordinates and the binding free-energy values of each minimum are reported in Table S1. A cluster analysis of the conformations belonging to each minimum hole was then performed (see the Methods section for details). In each minimum, the first cluster is the most populated (Figure S10), and its centroid was used as the representative structure for that minimum. Looking at the FES (Figure 4), three different regions can be identified following CV1: the bound state, with *s*(*R*) values between 1 and 3 (minima A–C), the intermediate states, with *s*(*R*) values between 3 and 7 (minima D–G), and the unbound state, with *s*(*R*) values from 7 onward. In the deepest minimum (A), the ligand is oriented in the same way as in the X-ray structure (ligand RMSD = 1.36 Å). This geometry is stabilized by a hydrogen bond between the NH group of the ligand and the H248 residue as well as by a transient hydrogen bond between the oxygen atom of furan and the S246 residue.

Moving up to higher CV2 values, alternative binding geometries can be detected. In minimum B, the ligand is shifted toward the exit of the cavity, and breaking of the hydrogen bonds that stabilize minimum A causes a lower stability. Instead, in minimum C, the ligand is located on the mouth of the binding cavity and is even turned 180°, with the CF_3_ group oriented toward the bottom of the cavity. Also, in this minimum, the amino group of the ligand forms a hydrogen bond with the S292 residue. This last conformation represents the second minimum in energy.

### Unbinding Pathway for the KG-721 Ligand

Following the encouraging results on THS-020, for which the selected methods were able to identify the experimental binding geometry as the most stable among all the possible bound states, we extended the study to the lower-affinity KG-721 ligand. Given the lack of an experimental structure, we obtained the starting geometry for our calculations by molecular docking, using the ensemble-docking technique^5^ (details are reported in the Methods section). This technique has led to improvement of the description of ligand-induced protein conformational changes in many systems,^4−7^ but it may be not sufficient in some particularly challenging cases. These include docking studies of ligands different from the ones cocrystallized in the protein structures of the ensemble (if any), like in our case.

As for the THS-020 ligand, 50 independent replicas of SMD simulations (each of 25 ns) were performed for the two possible pathways. The resulting work profiles (Figure S11) are similar to those obtained for THS-020. However, different from that ligand, they show a similar range of work values for path 1 and path 2 and do not suggest any preference for one path over the other. This is also confirmed by negligible differences between the values of *W*_min_, *W*_max_, and Δ*F*_unbind_ (Table 2) in the two paths. This result suggests that for a small ligand, the two pathways may have a similar probability. This hypothesis is consistent with the findings of Key et al.,^53^ who observed a similar percentage of transferring of the small water molecules in the two paths. However, the observed difference of 105.08 pN between the *F*_max_ values of the two paths of KG-721 suggests that a higher barrier for unbinding exists along path 2, similarly to what was observed for the THS-020 ligand.

**Table 2:** Results of SMD Simulations for the KG-721 Ligand

pathway	*W*_min_ (kcal/mol)	*W*_max_ (kcal/mol)	*F*_max_ (pN)	Δ*F*_unbind_ (kcal/mol)	st. dev.
1	28,46	54,45	851,21	30,55	13,04
2	27,47	59,13	956,29	27,12	16,60

Based on the results obtained with the steered MD simulations, 11 frames along path 1 were used to build the reference path for metadynamics simulations. The resulting RMSD matrix of the frameset and a representation of the reference path are shown in Figure S12.

After 3 μs of metadynamics simulation, we reconstructed the free-energy profile (Figure S13a). Starting from 2200 ns, the free-energy difference between the bound and the unbound states fluctuates around a value of −8.0 kcal/mol with a variation of ±1 kcal/mol. The calculated binding free energy revealed that the KG-721 ligand has a lower binding affinity than THS-020 (−11.8 kcal/mol), in agreement with the experimental data: Δ*G*_exp_ (KG-721) = −6.9 ± 0.1 kcal/mol and Δ*G*_exp_ (THS-020) = −7.9 ± 0.5 kcal/mol.

During the simulation, we observed multiple binding and unbinding events, and the hill heights decrease toward 0 (Figure S13b,c).

**Figure 5:**
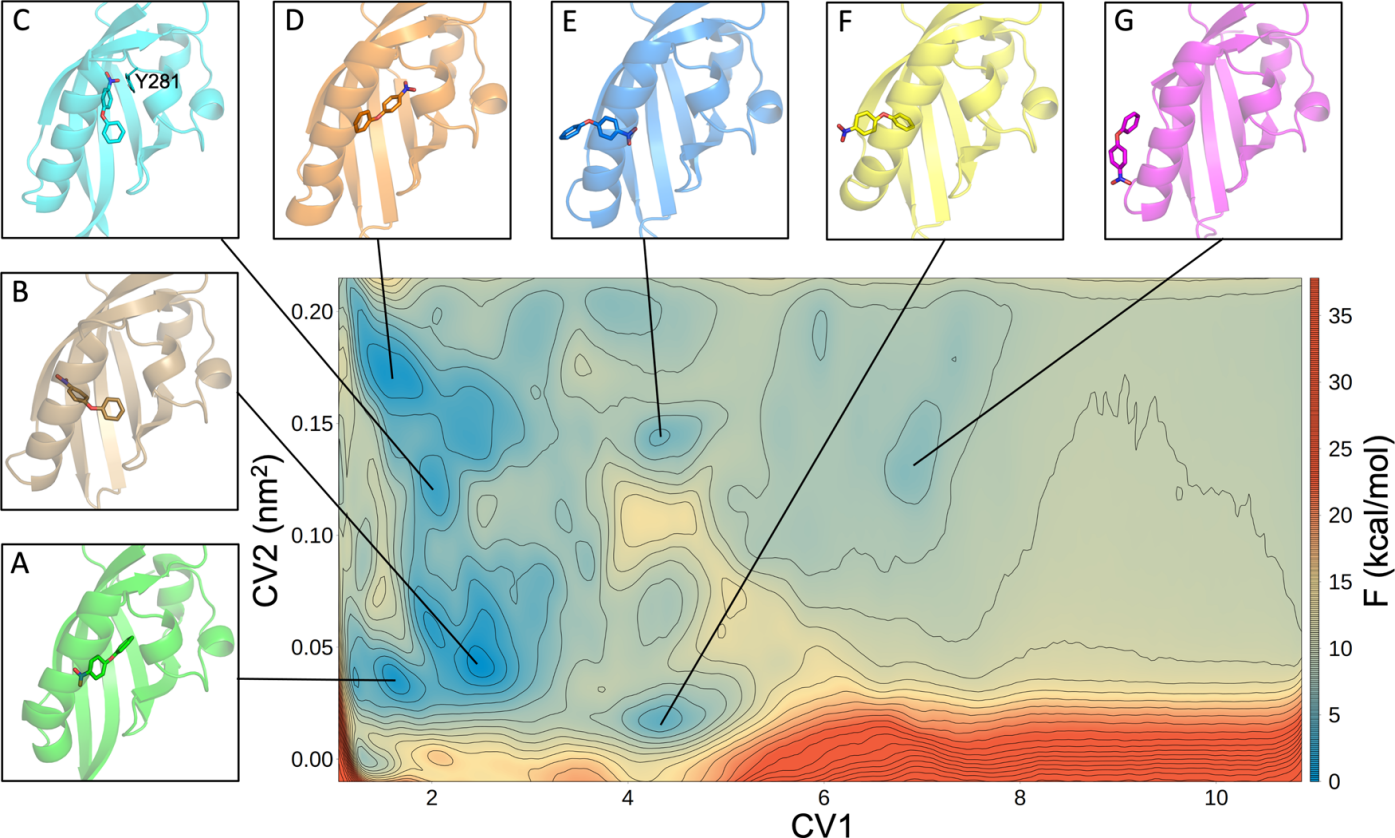
Free-energy surface obtained from the PCV approach for the binding/unbinding of the KG-721 ligand. The isolines are drawn using a 1.5 kcal/mol spacing. The 3D structures of the centroids of the main minima are reported with different colors: the protein is represented as cartoons and the ligand as sticks. The black lines indicate the corresponding minima in the FES.

The final FES obtained for this system is shown in Figure 5. Even in this case, we identified several minima (details in Table S1), and we used the centroid of the most populated cluster (details on cluster compositions in Figure S14) in each minimum, as the representative structure of that minimum.

**Figure 6:**
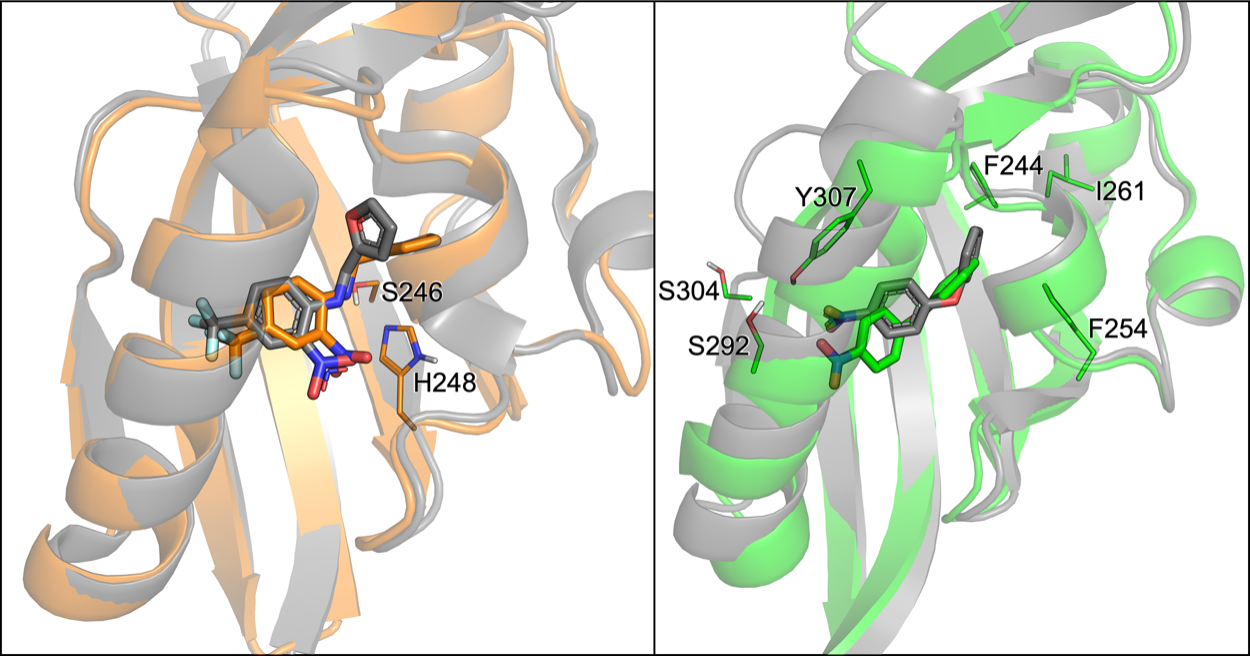
Comparison between minimum A and the starting structure for the two ligands. On the left: for the THS-020 ligand, the overlay of minimum A, in orange, with the crystallographic structure of the complex, in gray. On the right: for the KG-721 ligand, the overlay of minimum A, in green, with the docking pose, in gray.

Again, following the CV1, three regions can be distinguished: the bound state, between 1 and 3, the intermediate states, between 3 and 7, and the unbound state, from 7 and on. The region around the bound state displays a multiplicity of alternative binding geometries and does not allow us to distinguish a favorite bound minimum. Minima from A to D can be associated to alternative bound states in which the ligand rotates within the binding cavity. In particular, in minimum A, the ligand is oriented with NO_2_ toward the most polar part of the cavity (S292, S304, and Y307 residues, at the entrance of the cavity) and the phenyl ring toward the apolar part (F244, F254, and I261 residues, at the bottom of the cavity), as expected (Figure 6, right panel). Even in minimum B, NO_2_ is oriented toward the polar region, but the phenyl ring is slightly bent with respect to the other ring. Moving up to higher CV2 values, minima present different orientations of the ligand inside the cavity: minimum C is stabilized by a hydrogen bond between NO_2_ and the Y281 residue; in minimum D, the ligand is rotated 180° with respect to minimum A and does not show stable hydrogen bonds with the protein.

While we previously observed that in the deepest minimum (A), THS-020 well overlaps the experimental binding geometry (Figure 6, left panel), minimum A of KG-721 (Figure 6, right panel) is the most similar to the starting docking pose (where values in the CV subspace are *s*(*R*) = 1.3 and *z*(*R*) = 0). Indeed, the RMSD between the centroid of minimum A and the docked pose is 2.45 Å, indicating that the two conformations are quite different.

In the case of KG-721, which is not congeneric with any of the cocrystallized HIF-2α ligands,^50,51,53^ the ensemble-docking strategy was not sufficient for the correct definition of the binding mode. In light of our results, we underline the importance of including protein flexibility more completely. Our results indicate that MetaD calculations are not influenced by the inaccurate starting conformation of the complex but lead the system to evolve to a more stable conformation. Therefore, this technique appears a promising tool in cases where structural information for congeneric ligands is not available.

## Conclusions

Modeling the pathways for ligand binding to the HIF-2α PAS-B domain represents a nontrivial task due to the buried nature of the binding cavity that suggests that significant protein conformational changes may occur upon ligand access. The computational protocol here proposed effectively combines two promising methods based on enhanced-sampling MD. Steered MD simulations are used to identify the preferred unbinding pathway among alternative ones and to guide the construction of the reference path for the subsequent step. On the other side, metadynamics, with the path collective variable formalism, is used to obtain a more rigorous characterization of the free-energy surface and to calculate the binding free-energy value.

By applying this approach to elucidate the binding process of two different ligands of HIF-2α, we obtained the correct binding affinity scale, according to the experimental data available, and we identified minima in the FES that clearly depict the bound state(s) and the intermediate states characteristic of each ligand. Moreover, the method was effective in leading the system to evolve to the most stable binding conformation, starting either from an X-ray structure of the ligand-protein complex or from a docking pose. Therefore, it appears a promising tool also in cases where reference structural information is lacking.

Given the recent discovery of HIF-2α as a pharmaceutical target for cancer therapy, the proposed computational approach based on enhanced-sampling MD appears to be an invaluable tool to investigate the binding process of different ligands, thus contributing to the development of successful drug design projects. The results obtained here also encourage us to extend applications to other binding mechanisms of bHLH-PAS proteins, including significant targets, such as the AhR, for which no experimental structural information on the ligand-bound states is available.

## Supporting Information Available

(Supplementary text) Construction of path CVs, (Table S1) coordinates and binding free-energy values of the relevant minima along the CV1 (*s*(*R*)) and CV2 (*z*(*R*)), (Figure S1) plot of the RMSD values computed on Cα atoms for the HIF-2α PAS-B domain during the unbiased MD simulation, (Figure S2) representation of the pulling variable for the two paths, (Figure S3) selection of protein atoms for RMSD calculations, (Figure S4) monitoring of the conformational changes during unbiased MD simulation of the HIF-2α PAS-B with the THS-020 ligand, (Figure S5) plots of the RMSD values computed on Cα atoms for the THS-020 SMD replicas along path 1, (Figure S6) DSSP plots for the HIF-2α secondary structure transitions, (Figure S7) work profiles for the two unbinding pathways of THS-020, (Figure S8) resulting reference path for PCVs for the THS-020 ligand, (Figure S9) plot of the hill heights during the metadynamics simulation for the THS-020 ligand, (Figure S10) cluster analysis on each minimum of the THS-020 FES, (Figure S11) KG-721 unbinding pathways, (Figure S12) resulting reference path for PCVs for the KG-721 ligand, (Figure S13) results of KG-721 MetaD simulation, and (Figure S14) cluster analysis on each minimum of the KG-721 FES (PDF)

## Abbreviations

MD: molecular dynamics
SMD: steered molecular dynamics
MetaD: metadynamics
PMF: potential of the mean force
CVs: collective variables
FES: free-energy surface
PCVs: path collective variables
HIF-2α: hypoxia-inducible factor 2α
ARNT: aryl hydrocarbon receptor nuclear translocator
bHLH-PAS: basic helix–loop–helix/Per-ARNT-SIM
AhR: aryl hydrocarbon receptor
RMSD: root-mean-square deviation
