# Supplementary material for: Metadynamics-Based Approaches for Modeling the Hypoxia-Inducible Factor 2α Ligand Binding Process
Source: J Chem Theory Comput. 2021 Jun 4;17(7):3841–51. doi: 10.1021/acs.jctc.1c00114 (PMC8280741; doi:10.1021/acs.jctc.1c00114)
Supplement: Supplementary file 1 — ct1c00114_si_001.pdf [file ct1c00114_si_001.pdf]

Metadynamics-based approaches for modeling the HIF-2α ligand binding process

*Lara Callea, Laura Bonati, Stefano Motta**

Department of Earth and Environmental Sciences, University of Milano-Bicocca, Piazza della Scienza 1, 20126 Milan, Italy

SUPPLEMENTARY TEXT

Construction of Path CVs

The reference path was created for each ligand starting from the SMD simulation with the lowest value of the unbinding work. Using an in-house developed script implemented in VMD[1], the RMSD matrix was calculated for a selection of protein atoms (Figure S2) and all the ligand heavy atoms.

The optimal reference path should have a regular symmetric matrix (with a typical gull-wing shape)[2]. Anyway, in SMD simulations, when the ligand reaches the unbound state, it freely moves in the solvent thus providing an inhomogeneous frame-to-frame distance, and an irregular matrix. To avoid this problem, only the frames belonging to the first part of the path (ligand in contact with the protein) were extracted from the SMD simulations. Starting from the first frame (F, ligand in the bound state), equally spaced frames with a distance of 2 Å from the previous one were selected. In the last frame of this part (M) the ligand is located near to the mouth of the cavity. The ligand was then translated in the bulk solvent (frame L) and the second part of the path was obtained by a 2 Å linear interpolation between frames M and L. The combination of the frames selected from the SMD simulation and those obtained with linear interpolation provided the reference path.

SUPPLEMENTARY TABLES

Table S1. Coordinates and binding free-energy values of the relevant minima along the CV1 (s(R)) and CV2 (z(R)) for the THS-020 and KG-721 ligands.

THS-020				KG-721			
MIN	s(R)	z(R) nm^2	ΔF (kcal/mol)	MIN	s(R)	z(R) nm^2	ΔF (kcal/mol)
A	1.214	-0.003	0.00	A	1.566	0.032	1.15
	1.360	0.017			1.699	0.038	
B	2.691	0.042	5.65	B	2.421	0.040	0.00
	3.019	0.056			2.553	0.046	
C	1.922	0.124	2.50	C	1.978	0.115	2.83
	2.146	0.136			2.089	0.129	
D	3.006	0.088	5.78	D	1.511	0.168	1.18
	3.209	0.111			1.699	0.175	
E	4.872	0.136	4.57	E	4.097	0.134	5.20
	5.583	0.165			4.752	0.153	
F	5.262	0.024	6.91	F	4.244	0.015	4.40
	5.411	0.029			4.401	0.021	
G	6.694	0.084	7.63	G	6.815	0.124	6.15
	6.772	0.098			7.008	0.143	

SUPPLEMENTARY FIGURES

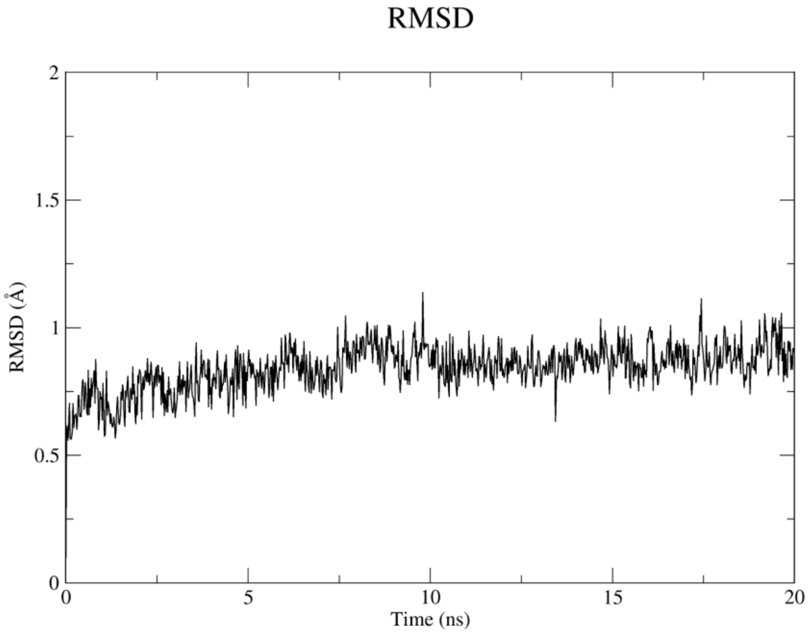

Figure S1. Plot of the RMSD values computed on Cα atoms for the HIF-2α PAS-B domain during the unbiased MD simulation.

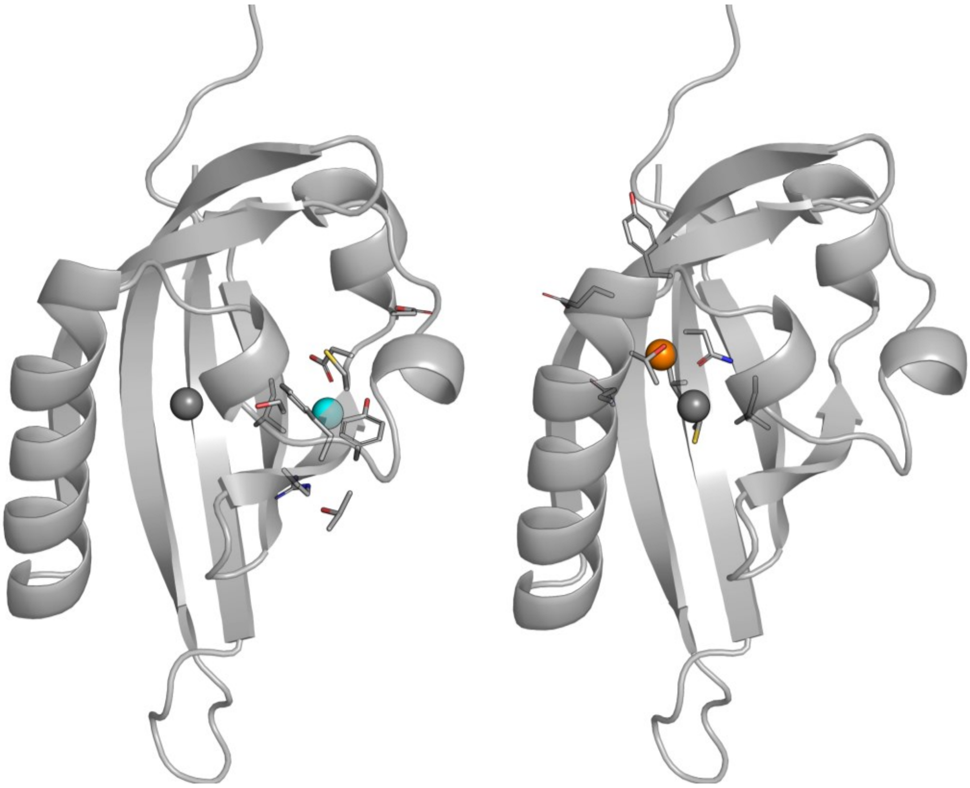

Figure S2. Representation of the pulling variable for the two paths (path 1 on the left, path 2 on the right). In cartoon, the PAS-B domain of HIF2α; in gray sphere, the center of mass of the heavy atoms of the ligand (THS-020); in cyan (orange) sphere, the center of mass of N, Cα, C and O atoms of selected reference residues: for path 1, L245, S246, R247, F254, T255, Y256, C257, D258, D259; for path 2: L245, E320, T321, Q322, G323, C339, V340, N341, Y342.

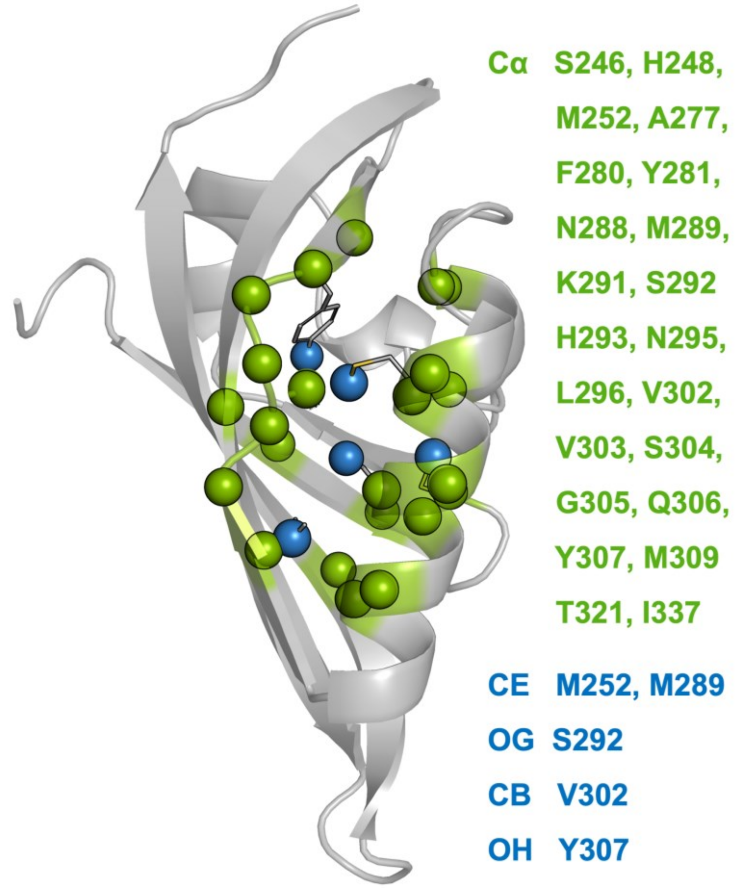

Figure S3. Selection of protein atoms for RMSD calculations. In green spheres, the Cα atoms; in blue spheres, some sidechain atoms.

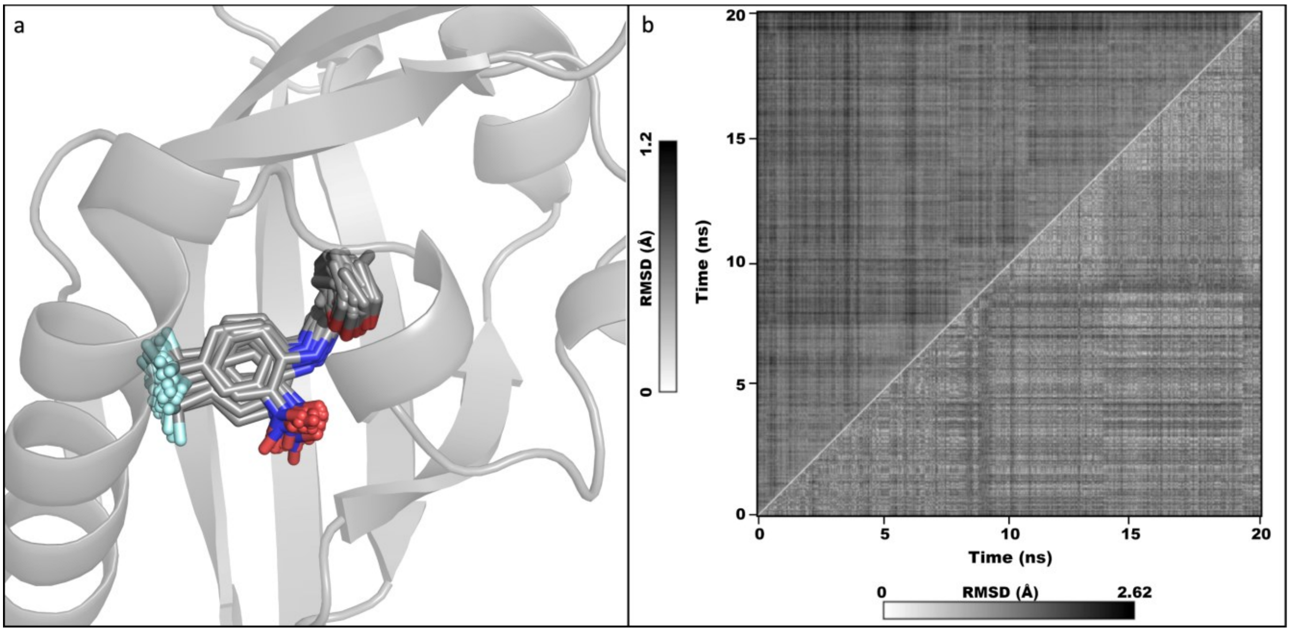

Figure S4. Monitoring of the conformational changes during unbiased MD simulation of the HIF-2α PAS-B with the THS-020 ligand. a) Representation of the ensemble of 50 ligand conformations in the last 10 ns of

simulation. The protein structure is represented as cartoons and the different states of the ligand as sticks.

b) RMSD matrix computed on Cα atoms (upper half) and on ligand heavy atoms (lower half).

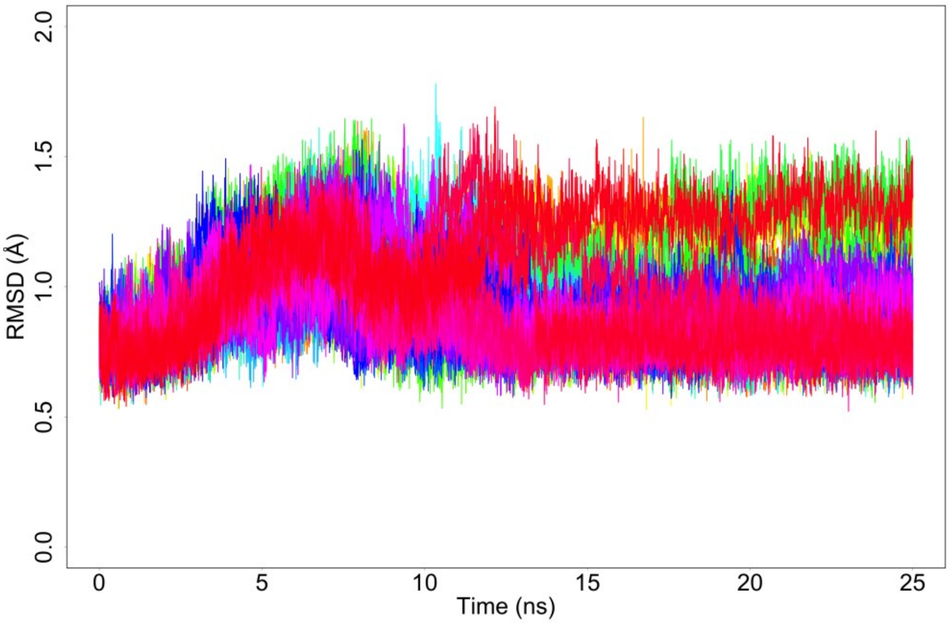

Figure S5. Plots of the RMSD values computed on Cα atoms for the THS-020 SMD replicas along path 1.

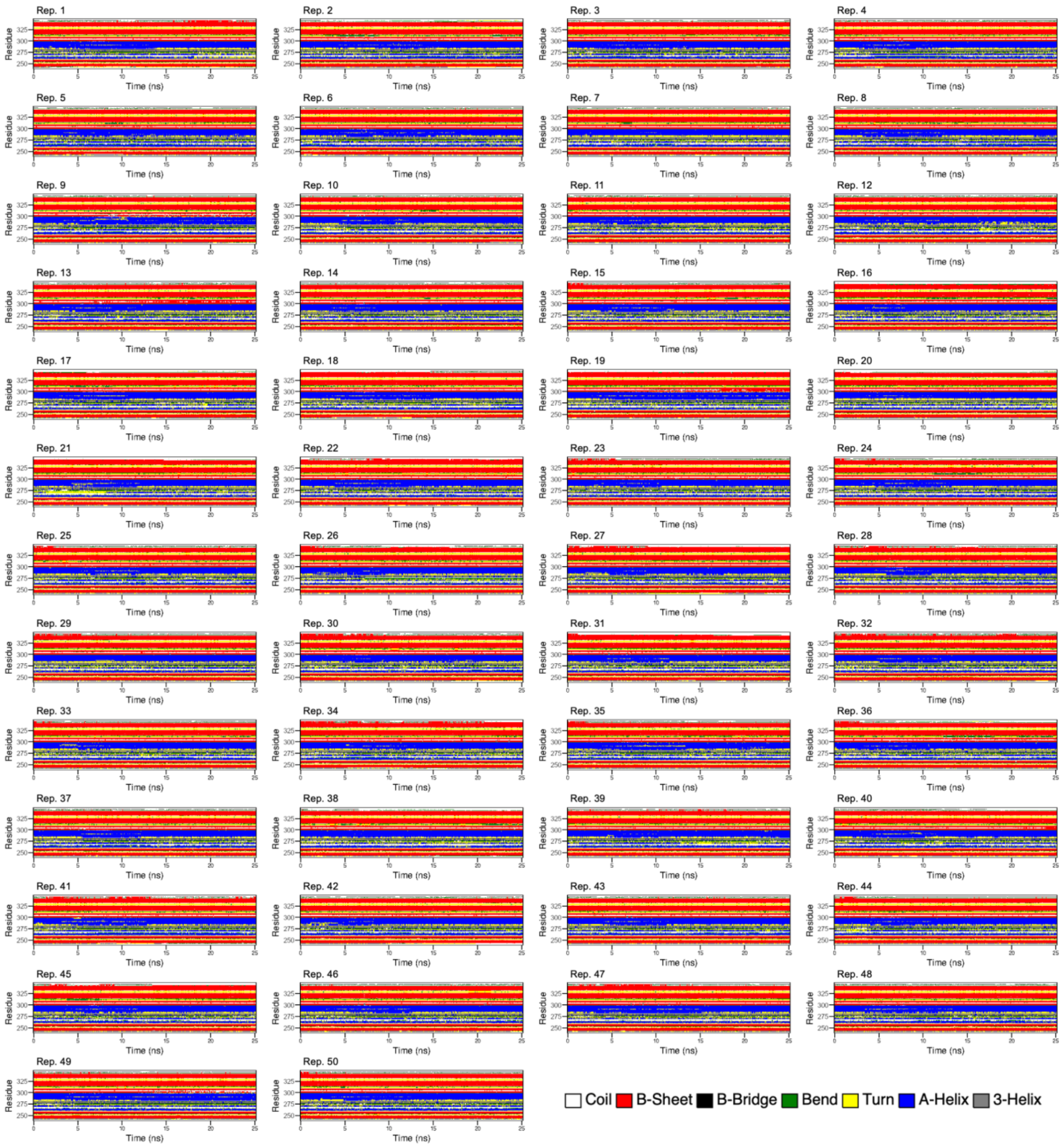

Figure S6. DSSP plots for the HIF-2α secondary structure transitions during the THS-020 SMD replicas along path 1.

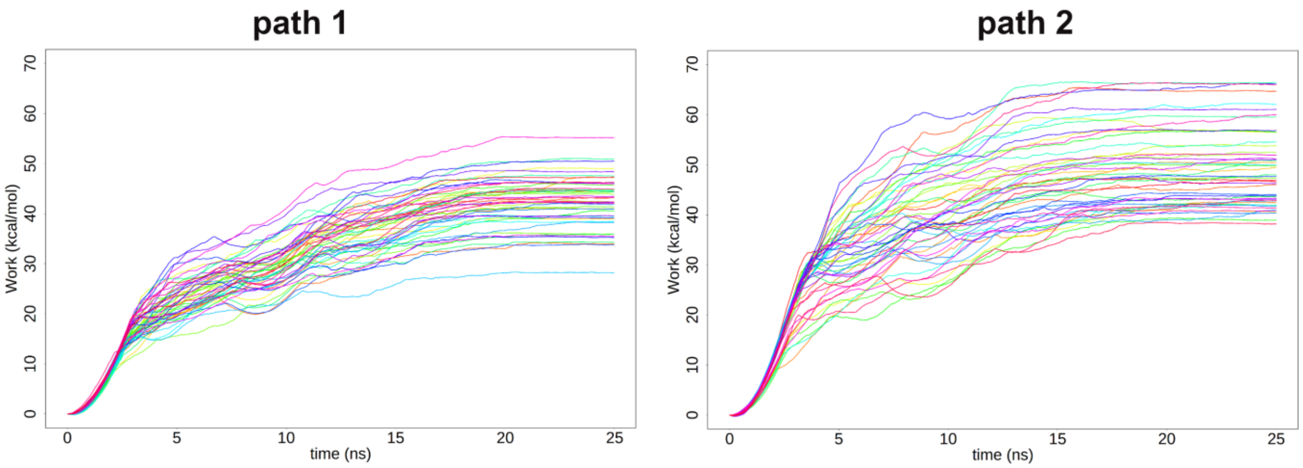

Figure S7. Work profiles for the two unbinding pathways of THS-020: the 50 curves of the work exerted on the system to pull the ligand along path 1, on the left, and along path 2, on the right.

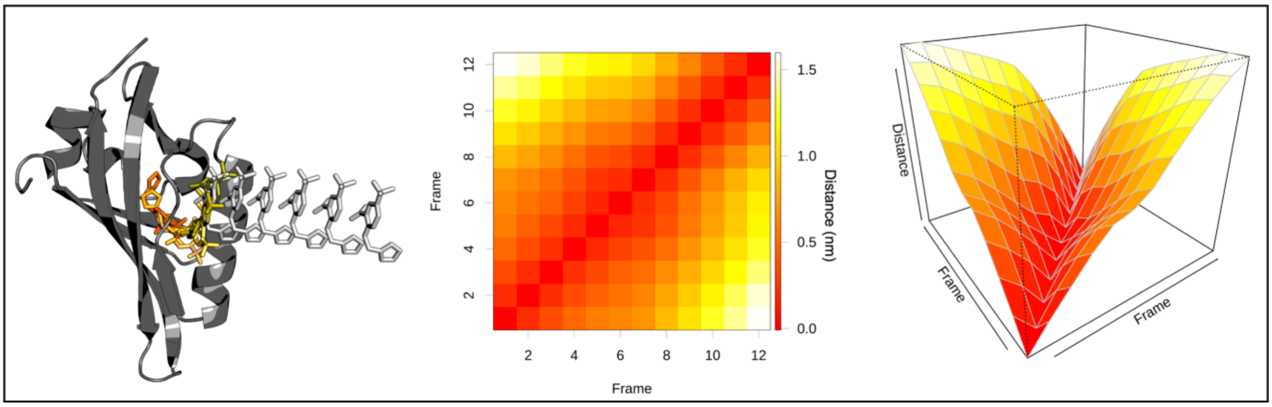

Figure S8. Resulting reference path for PCVs for the THS-020 ligand. Protein is represented in the bound conformation as dark grey cartoons, the ligand in the first part of the path (frames from SMD) as sticks from orange to olive, and the ligand in the second part of the path (frames extrapolated from linear interpolation) as light grey sticks. 2D (center) and 3D (right) representation of the RMSD matrix obtained from the frameset built for the THS-020 ligand.

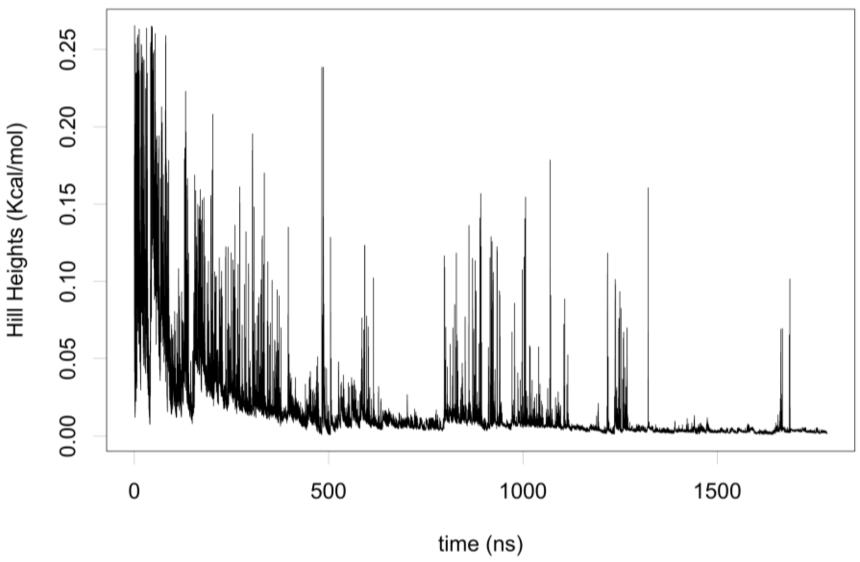

Figure S9. Plot of the hill heights during the metadynamics simulation for the THS-020 ligand.

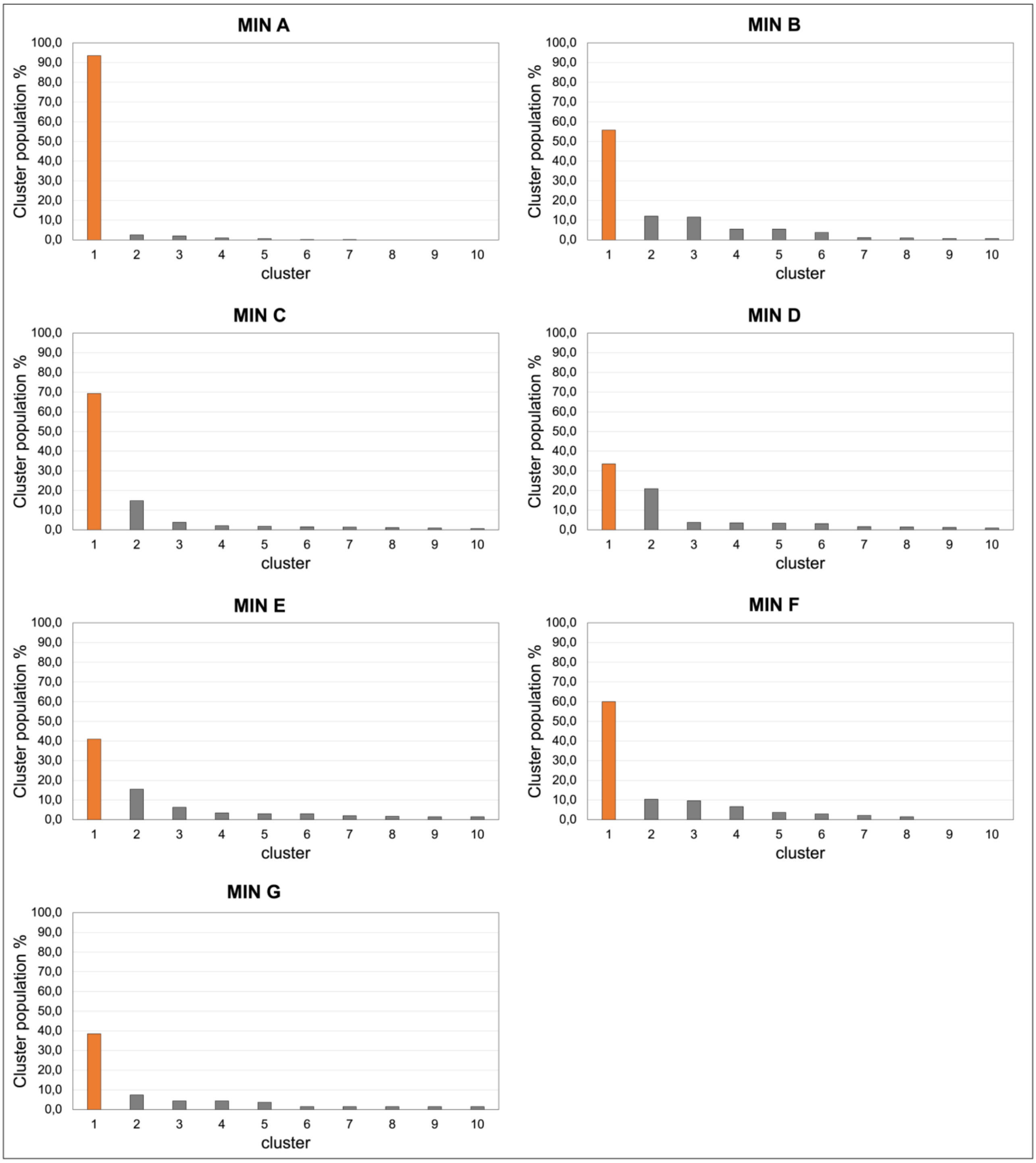

Figure S10. Cluster analysis on each minimum of the THS-020 FES: Barplot representation of the cluster population for the first 10 clusters of each minima.

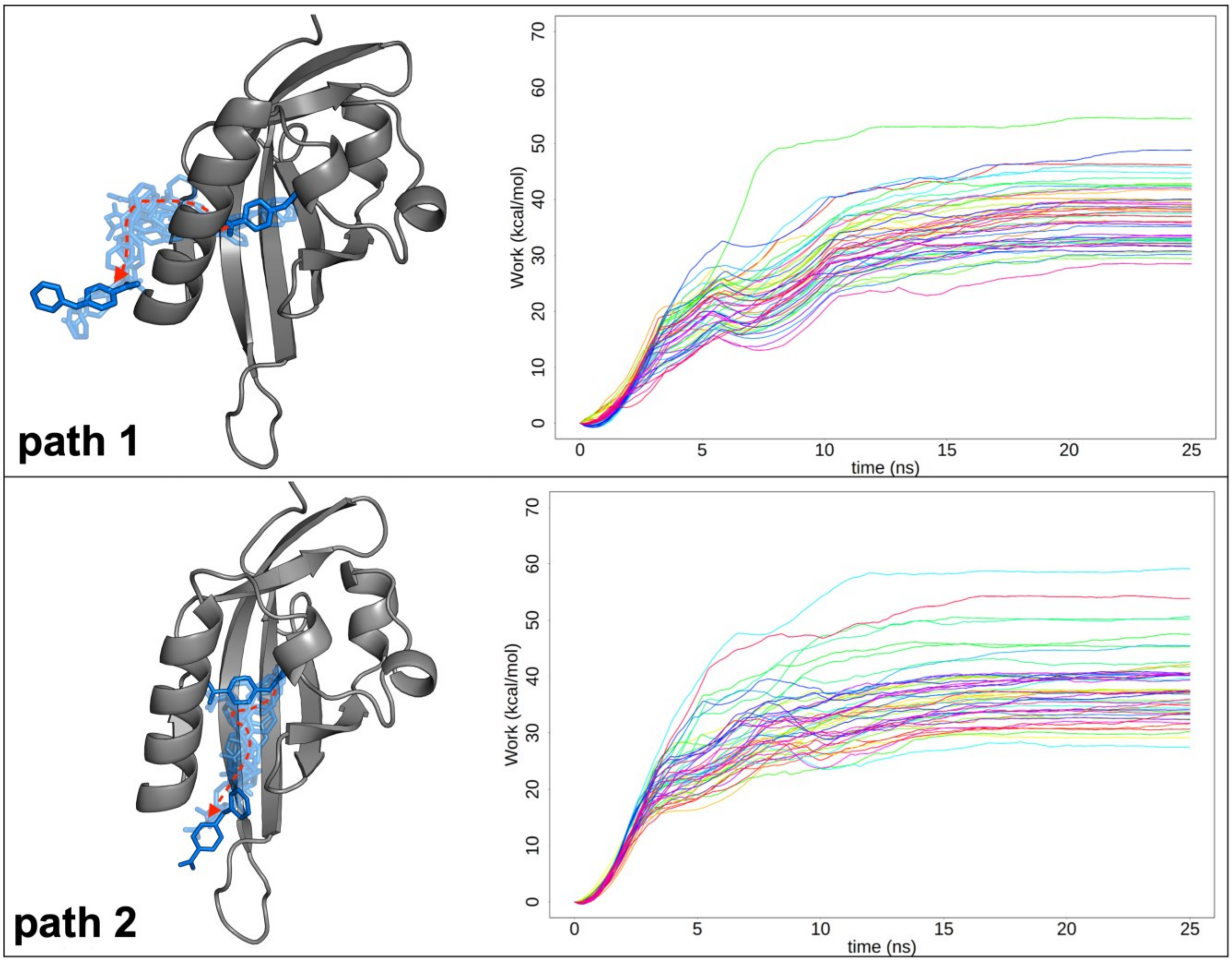

Figure S11. KG-721 unbinding pathways. In pathway 1 (above) the ligand passes through Fα and Gβ, while in pathway 2 (below) through Fα, Eα and the AB loop. The starting protein structure is represented as grey cartoons, the ligand conformations in the first and last frames of the trajectory as blue sticks, and the conformations of the ligand in the intermediate frames as transparent sticks. On the right, the work profiles for the two paths.

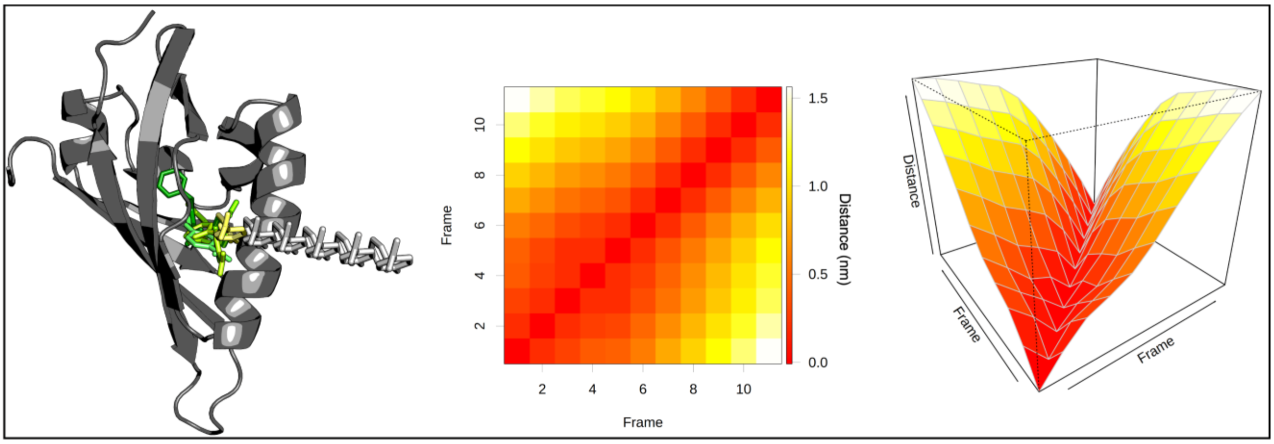

Figure S12. Resulting reference path for PCVs for the KG-721 ligand. Protein is represented in the bound conformation as dark grey cartoons, the ligand in the first part of the path (frames from SMD) as sticks from green to limon, and the ligand in the second part of the path (frames extrapolated from linear interpolation) as light grey sticks. 2D (center) and 3D (right) representation of the RMSD matrix obtained from the frameset built for the KG-721 ligand.

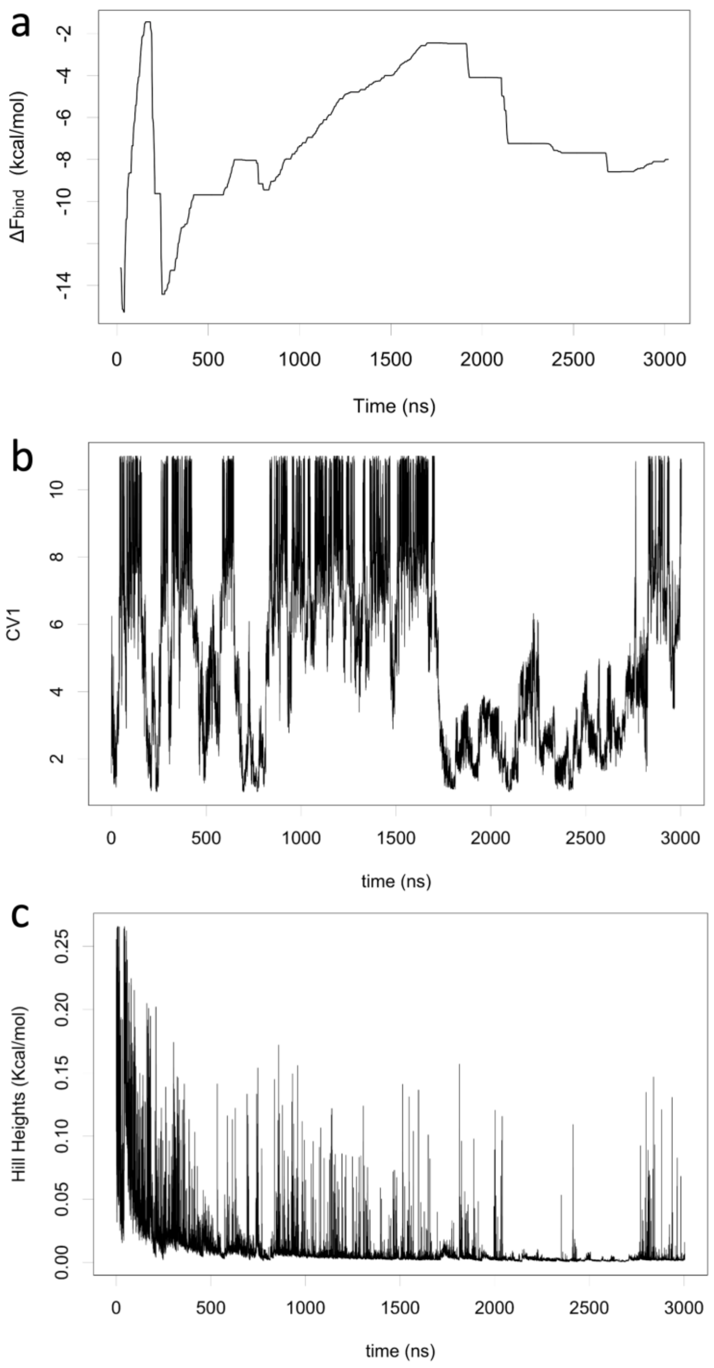

Figure S13. Results of KG-721 MetaD simulation. a) one-dimensional projection of the binding free-energy values associated to the path 1; b) instantaneous values of CV1 (s(R)); c) plot of the hill heights during the simulation time.

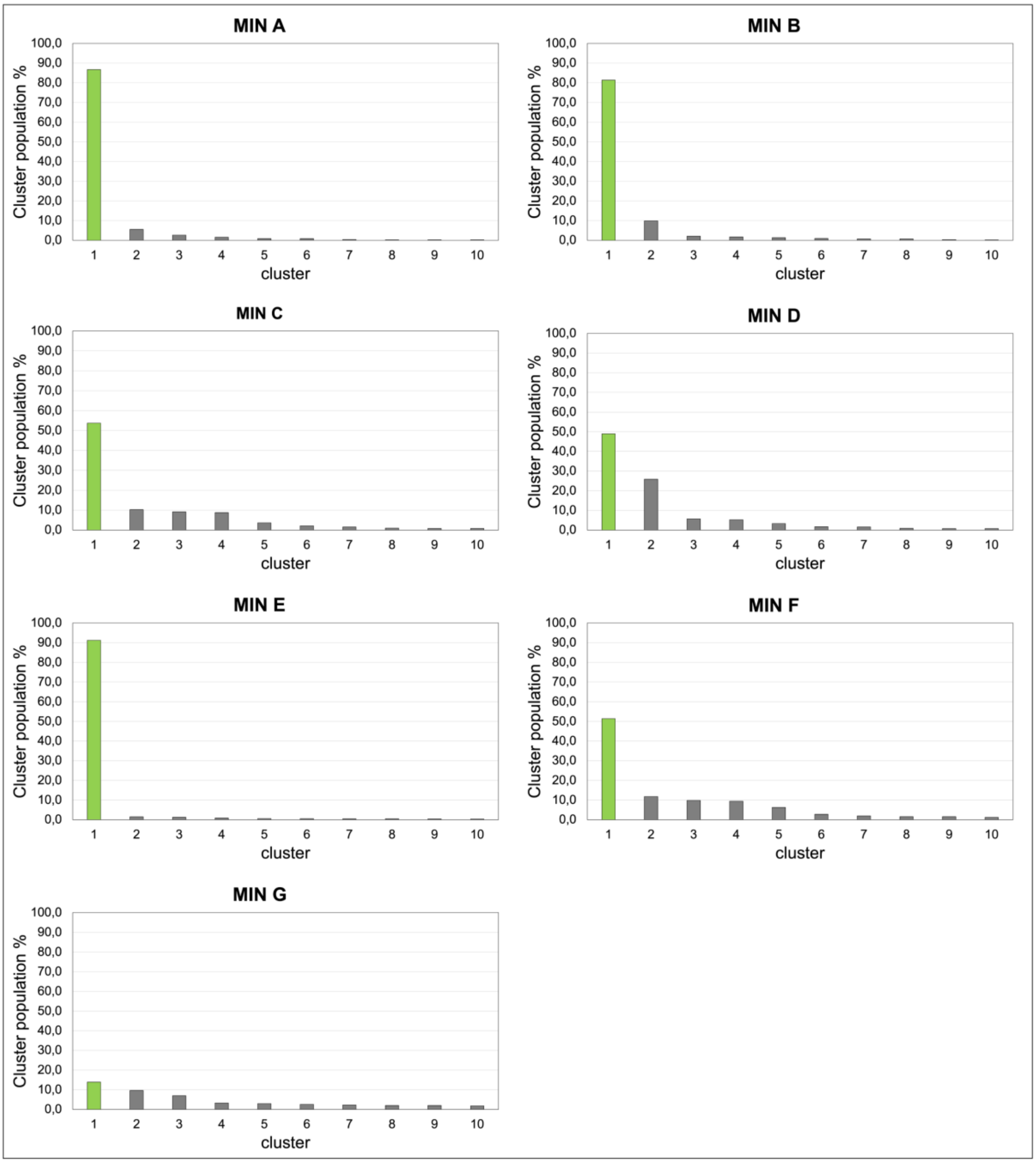

Figure S14. Cluster analysis on each minimum of the KG-721 FES: Barplot representation of the cluster population for the first 10 clusters of each minima.

1. Humphrey, W., Dalke, A. & Schulten, K. VMD: Visual molecular dynamics. *J. Mol. Graph.* 14, 33–38 (1996).

2. Branduardi, D., Gervasio, F. L. & Parrinello, M. From A to B in free energy space. *J. Chem. Phys.* 126, 054103 (2007).
